# Comparative Assessment of Manual Versus Automated Semen Analysis and the Role of Novel Biomarker Calprotectin in Screening for Male Factor Infertility

**DOI:** 10.22034/ijp.2026.2087408.3647

**Published:** 2026-08-01

**Authors:** Aiswarya AB, Mukta Pujani, Nishant Gurnani, Varsha Chauhan, Sonam Bhatia, Mitasha Singh, Charu Agarwal, Garima Dhull, Meenu Pujani

**Affiliations:** 1 Department of Pathology, Urology, and Biochemistry, ESIC Medical College & Hospital, Faridabad, Haryana, India; 2 Department of Community Medicine, Dr. Baba Saheb Ambedkar Medical College and Hospital, Delhi, India; 3 Department of Lab Services, Metro Heart Institute & Multispecialty Hospital, Faridabad, Haryana, India

**Keywords:** manual semen analysis, computer-assisted sperm analysis, CASA, calprotectin, infertility

## Abstract

**Background & Objective:**

Although manual semen analysis (MSA) is cost-effective and widely used, it is prone to subjectivity and operator variability, prompting the need for standardized, automated systems such as computer-assisted semen analysis (CASA). This study was conducted to compare semen parameters using manual and automated methods and assess the relationship between calprotectin levels and semen quality.

**Methods:**

This prospective cross-sectional study was conducted over a period of 1 year. A total of 200 semen samples from male partners of infertile couples were evaluated by manual as well as automated methods (MES SQA Vision). Calprotectin in seminal plasma was estimated by ELISA. Pearson correlation was used to assess associations, and paired t test was used to compare measurements. Agreement and systematic bias were evaluated using a Bland-Altman plot.

**Results:**

A strong correlation was observed between MSA and CASA for total sperm count (r = 0.950), concentration (r = 0.954), motility parameters (rapidly and slowly progressive motility) (r = 0.753 and 0.673), and vitality (r = 0.854), while nonprogressive motility showed a moderate correlation (r = 0.437). Smoking showed weak negative correlations with sperm count (r = –0.0073), concentration (r = –0.01), and motility, and a weak positive correlation with abnormal morphology. Alcohol showed weak positive correlations with count and concentration, but weak negative effects on motility and morphology. Semen calprotectin showed weak negative correlations with sperm count (r = –0.04052), concentration (r = –0.03939), motility (r = –0.16466), and morphology (r = –0.09623) and a weak positive correlation with vitality (r = 0.06115).

**Conclusion:**

The computer-assisted sperm analysis (CASA) system represents a significant advancement in reproductive diagnostics by offering precise, objective, and reproducible assessment of key semen parameters, showing agreement with MSA for most of the parameters. Further studies with larger sample sizes are required to clarify the diagnostic significance of calprotectin, an inflammation-related biomarker in seminal plasma.

## Introduction

Infertility, defined as the inability to achieve pregnancy after 12 months of regular unprotected intercourse, affects around 15% of couples globally, with male factors contributing to 40–50% of cases(1). Male infertility may result from obstructive, hormonal, testicular, genetic, environmental, or lifestyle causes(2). Semen analysis remains the first-line investigation to assess sperm concentration, motility, morphology, vitality, and overall reproductive potential.

Although manual semen analysis (MSA) is widely used due to its cost-effectiveness, it is prone to subjectivity and operator variability, prompting the need for standardized, automated systems such as computer-assisted semen analysis (CASA), including the MES SQA Vision, which enhances accuracy, reproducibility, and efficiency(3).

In addition to semen parameters, seminal plasma biomarkers are increasingly explored for their diagnostic potential, with calprotectin—a calcium-binding inflammatory marker—but limited studies exist regarding its role in male infertility(4).Therefore, the present study aims to compare manual and automated semen analysis methods and evaluate calprotectin as a novel biomarker for screening male infertility(4).

This study was conducted to compare semen parameters (concentration, motility, vitality, morphology) using manual methods and an automated method, correlate semen parameters with clinicopathological factors (e.g., smoking, alcohol use, occupation, infections), estimate seminal plasma calprotectin levels, and assess the relationship between calprotectin levels and semen quality.

## Materials and Methods

This was a prospective cross-sectional study conducted in the Department of Pathology at ESIC Medical College & Hospital, Faridabad, over a period of 1 year from August 31, 2023. The study was approved by the Institutional Ethics Committee. Written informed consent was obtained from all participants.

A total of 200 semen samples were collected from male partners of infertile couples attending the infertility clinic. The minimum required sample size (n = 196) was calculated using a 15% prevalence of infertility in India, 95% confidence interval, and 5% absolute error.

Male partners of infertile couples presenting to the infertility clinic were included in the study. Patients with semen volume <1 mL and patients unwilling to participate in the study were excluded.

Participant information on smoking, alcohol use, occupation, infections, sexually transmitted diseases, drug intake, and trauma was recorded via a prestructured proforma.

### Semen Collection and Analysis (Manual Method)

Semen samples were collected by masturbation after 2–7 days of sexual abstinence. Samples liquefied at 37°C and were analyzed within 60 minutes. Semen volume, viscosity, pH, sperm concentration, motility (rapidly progressive, slowly progressive, nonprogressive, and immotile), vitality (eosin-nigrosin staining), and morphology (by Papanicolaou staining) were evaluated.

### Automated Semen Analysis (SQA Vision)

Sperm parameters such as concentration, motility, vitality, and morphology were also assessed using the MES SQA Vision automated semen analyzer, which uses light absorption and motion analysis algorithms for parameter quantification.

Following semen analysis, the samples were centrifuged, and seminal plasma was collected for estimation of calprotectin levels using a standardized biochemical assay, ELISA.

### Statistical Analysis:

Statistical analysis was conducted using SPSS version 23 (IBM). Sperm concentration, motility, and morphology were expressed as mean ± standard deviation (SD). Pearson correlation was used to assess associations, and paired t test was used to compare measurements. Agreement and systematic bias were evaluated using a Bland-Altman plot. A P value < 0.05 was considered statistically significant.

## Results

The study comprised a total of 200 participants with a mean age of 30.87 ± 5.18 years. Semen pH values (8.0–8.5) and liquefaction times (20–60 minutes) fell within expected physiological ranges. Among the 200 samples, 7.5% showed azoospermia, and 33.5% presented sperm concentrations between 40 and 100 million/mL, a range consistent with normal to high sperm density. A predominantly sedentary lifestyle was observed in 62.5% of participants, which is relevant given its association with obesity and hormonal disturbances.

Primary infertility accounted for 78% of cases, while 22% represented secondary infertility, indicating underlying differences in reproductive history. Table 1 depicts the clinicodemographic profile of the study population.

**Table 1:** Clinico-Demographic Profile of the study population (n=200)

No	Parameter	Subcategories	No. of cases	Percentage
1	Age Mean +SD= 30.87± 5.18	<30 year	90	45%
		> 30 year	110	55%
2	Occupation	Sedentary	125	62.5%
		Active	75	37.5%
3	Type of Infertility	Primary	156	78%
		Secondary	44	22%
4	Smoking	Present	66	33%
		Absent	134	67%
5	Alcohol	Present	47	23%
		Absent	153	77%
6	History of STD	Present	13	6%
		Absent	187	94%
7	History of Tuberculosis	Present	2	1%
		Absent	198	99%
8	History of Trauma	Present	17	8%
		Absent	183	92%
9	History of Undescented Testes	Present	6	3%
		Absent	194	97%
10	History of Drug Intake	Present	6	3%
		Absent	194	97%

### Comparison of Manual and Automated Analysis

**Fig. 1:**
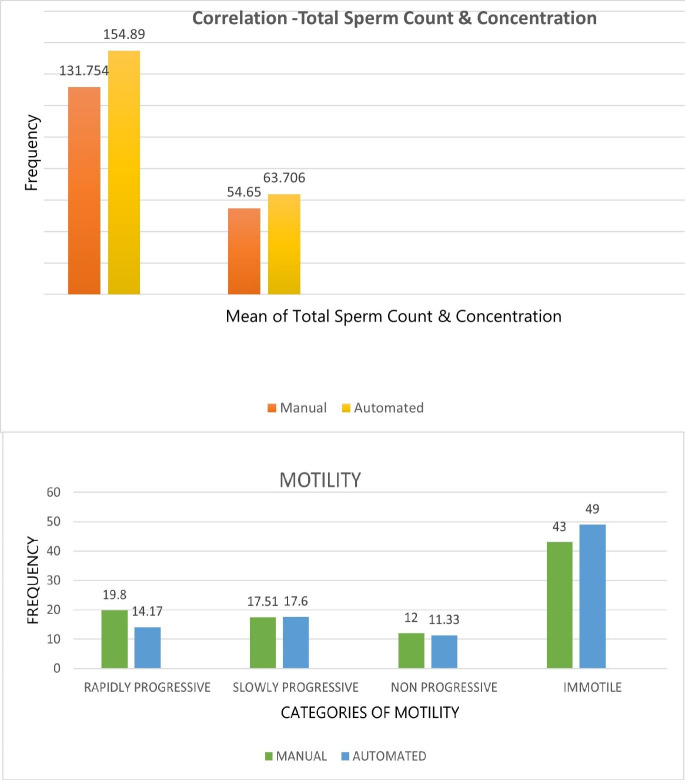
Graphic image showing A) Comparative evaluation of Sperm Count and Sperm Concentration by Manual vs Automated methods; B) Comparative evaluation of Rapidly Progressive Motility, Slowly Progressive Motility, Non-Progressive Motility and Immotile sperm using Manual vs Automated methods.

A strong correlation was observed between MSA and CASA for total sperm count (r = 0.950) and concentration (r = 0.954). Motility parameters (rapidly and slowly progressive motility) showed strong positive correlations (r = 0.753 and 0.673), while nonprogressive motility showed a moderate correlation (r = 0.437). Vitality also showed a strong correlation (r = 0.854) between methods (Tables 2 and 3; Fig. 1).

**Table 2:** Comparison between semen parameters on Manual vs Automated analysis

	Parameter	Subcategories	Manual	Automated	p value
1	Total count		131.754±140.87	154.89±187.44	0.001
2	Concentration		54.65±50.19	63.706±60.28	0.000
3	Motility	Rapidly Progressive	19.8±15.93	14.17±13.47	0.000
		Slowly Progressive	17.51±10.72	17.6±11.5	0.925
		Non Progressive	12±6.95	11.33±9.27	0.085
		Immotile	43±21.77	49±22.8	0.000

**Table 3:** Comparison between sperm morphology parameters on Manual vs Automated analysis

Morphology	Normal Forms	Abnormal Head	Abnormal Mid piece	Abnormal tail	Excessive residual cytoplasm
Manual versus Automated	r = 0.656 p = 0.000	r = 0.528 p = 0.000	r = 0.388 p = 0.000	r = 0.371 p = 0.000	r = 0.186 p = 0.011

**Fig. 2:**
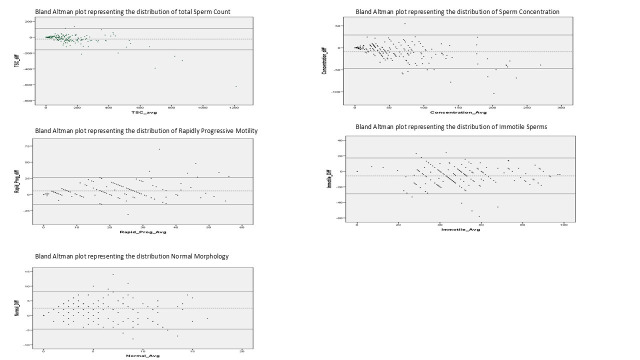
Bland–Altman plots for assessing agreement between manual semen analysis (MSA) and the Computer-Assisted Semen Analysis (CASA) system- Total Sperm Count, sperm Concentration, rapidly Progressive motility, Immotile sperms and Normal morphology.

To further evaluate the level of agreement between manual semen analysis (MSA) and the computer-assisted semen analysis (CASA) system, Bland-Altman plots were generated for the parameters that showed good correlation in Pearson analysis (Fig. 2). The Bland-Altman method assesses agreement by plotting the differences between 2 measurement techniques against their mean values, allowing identification of systematic bias, proportional bias, and the distribution of differences within the 95% limits of agreement (LoA).

For total sperm count, the Bland-Altman plot showed a mean difference of approximately –10 to –20, indicating a slight negative bias, where manual counts were marginally lower than CASA-derived values. The data points showed a narrow spread, and almost all values were within the 95% LoA, indicating good overall agreement. For sperm concentration, the mean difference ranged from –5 to –10, reflecting a small negative bias similar to total count, with manual measurements again slightly lower than automated values. Most points fell within the LoA with moderate scatter, demonstrating satisfactory agreement between the 2 methods.

For rapidly progressive motility, the mean difference was +5 to +10, indicating a positive bias where manual assessments tended to yield higher motility values than CASA. Although the data showed a wide spread, nearly all values fell within the LoA, supporting overall acceptable agreement, despite greater variability. For immotile sperm percentage, the mean difference ranged from –5 to –10, reflecting a negative bias where manual methods reported slightly lower immotile fractions than automated analysis. The plot showed wide scatter, with a few values outside the LoA, yet still suggested overall agreement between the 2 methods.

For normal morphology, the mean difference was approximately +2.5, showing a small positive bias, with manual assessment reporting marginally higher normal morphology than CASA. The plot displayed very wide variability, with several points outside the LoA, indicating only mild agreement between manual and automated morphology assessments.

Overall, Bland-Altman analysis confirmed that manual and automated semen analysis methods demonstrate good agreement for parameters such as total sperm count and concentration, acceptable agreement for motility indices, and limited agreement for morphology. These findings highlight the potential of CASA to complement or enhance manual semen analysis, while also emphasizing the need for caution when interpreting morphology results across different analytical platforms.

### Lifestyle and Clinical Correlations:

Smoking showed weak negative correlations with sperm count (r = –0.0073), concentration (r = –0.01), and motility, and a weak positive correlation with abnormal morphology. Alcohol showed weak positive correlations with count and concentration, but weak negative effects on motility and morphology, suggesting mild to moderate intake may not be detrimental. Sexually transmitted diseases (STDs) exhibited weak negative correlations with sperm count (r = –0.00171), confirming known adverse effects on fertility. Active occupational lifestyle was weakly positively correlated with sperm concentration and motility, while exposure to hazardous environments was known to impair fertility in other studies. Tuberculosis (1%) and drug use (3%) had too few cases for firm conclusions, though prior studies indicate potential harm to sperm quality (Table 4).

**Table 4:** Correlation of Semen parameters with smoking and alcohol intake

Parameter	Smoking		Alcohol	
	R value	Correlation	R value	Correlation
Total Sperm Count	-0.0073	Weak Negative	0.0043	Weak Positive
Concentration	-0.01	Weak Negative	0.015	Weak Positive
Rapidly Progressive	-0.001	Weak Negative	-0.009	Weak Negative
Slowly Progressive	-0.06	Weak Negative	-0.015	Weak Negative
Non progressive	-0.1	Weak Negative	0	No Correlation
Immotile	0.1	Weak Positive	0	No Correlation
Normal Morphology	0	No correlation	-0.1	Weak Negative
Abnormal Morphology	0.03	Weak Positive	0.0068566	Weak Positive

Calprotectin Analysis:

Semen calprotectin showed weak negative correlations with sperm count (r = –0.04052), concentration (r = –0.03939), motility (r = –0.16466), and morphology (r = –0.09623), and a weak positive correlation with vitality (r = 0.06115) (Table 5).

**Table 5:** Correlation between semen parameters and calprotectin

Parameter	R value
Total Sperm Count	−0.04052
Concentration	−0.03939
Motility	−0.16466
Normal Morphology	−0.09623
Vitality	+0.06115

## Discussion

Semen analysis remains the cornerstone in the evaluation of male infertility, providing essential information on sperm concentration, motility, morphology, and vitality. Manual semen analysis (MSA) continues to be widely used in most hospitals and ART laboratories due to its affordability and simplicity. However, its accuracy is highly operator-dependent. In contrast, computer-assisted semen analysis (CASA) offers automated, standardized, and reproducible assessment, reducing subjective variability and improving documentation efficiency.

In addition to comparing MSA and CASA, the present study also explored the influence of secondary clinical factors, such as lifestyle habits, systemic diseases, infections, occupational exposures, and biochemical markers—on semen parameters.

## Comparison of Manual and Automated Methods

The study demonstrated a strong positive correlation between MSA and CASA for both total sperm count and sperm concentration, which is consistent with studies by Agarwal et al., Finelli et al., Illardo et al., Engel et al., and Desole et al, showing CASA’s reliability for concentration measurements(5-9).

For motility parameters, immotile sperm percentage and rapidly progressive and slowly progressive motility showed strong correlations, while nonprogressive motility showed a moderate correlation. These findings are concordant with Robinson et al., Desole et al, and Illardo et al,(10,9,7) although some studies (Agarwal et al, Engel et al) have reported weaker agreement for progressive motility(5,8).

Vitality assessment also showed a strong correlation, although literature comparing vitality between manual and automated techniques remains limited.

## Correlation of Semen Parameters and Clinical Factors

Smoking demonstrated weak negative correlations with total sperm count, sperm concentration, and progressive motility parameters, along with a weak positive correlation with immotile sperm. These findings align with studies by Fan et al., Zhang et al., Tang et al, and Joo et al., which reported reductions in motility and concentration among smokers(11-14). Smoking showed no correlation with normal morphology but a weak positive correlation with abnormal morphology, consistent with Pereira et aland Cui et al, who reported increased morphological abnormalities and higher DNA fragmentation in smokers(15,16).

Alcohol showed weak positive correlations with total sperm count and sperm concentration—findings that differ from most studies reporting reduced semen quality (Muthusami et al., Bai et al.). However, these results align with Ricci et al, who observed improved semen parameters among moderate drinkers(17-19). Alcohol negatively affected progressive motility and normal morphology while slightly increasing abnormal morphology, consistent with Bai et al. and Joo et al., who reported deterioration in semen quality with alcohol consumption (18-14).

For STDs, a weak negative correlation with total sperm count was observed, similar to the findings from Fallahi et al., Davison et al., and Tsoumanis et al., who documented reduced semen quality in men with STDs(20-22).

Active occupational lifestyle showed a weak positive correlation with sperm concentration and progressive motility, with no correlation for nonprogressive or immotile sperm. Similar trends have been reported by Al-Otaibi et al, indicating that physically active occupations may support better semen quality compared with sedentary or high-heat exposure jobs(23). Although our data did not capture detailed exposure hazards, previous studies (Krausz et al, Mustafa et al, Rim et al) established strong evidence linking chemicals, radiation, and heat exposure with reduced fertility(24-26).

Calprotectin serves as an inflammatory biomarker in seminal plasma. In this study, calprotectin showed weak negative correlations with total sperm count, sperm concentration, motility, and normal morphology. Only vitality showed a weak positive correlation. These findings contrast with the only available reference study (Omes et al.), which reported higher calprotectin levels associated with better semen quality(4). No Indian studies have evaluated calprotectin in male infertility, highlighting the need for further research with larger cohorts to clarify its diagnostic value.

## Conclusion

The computer-assisted sperm analysis (CASA) system represents a significant advancement in reproductive diagnostics by offering precise, objective, and reproducible assessment of key semen parameters. Its high-throughput capability, ease of use, and enhanced analytical accuracy make CASA a valuable tool for clinical decision-making, treatment monitoring, and research applications in male reproductive health.

Lifestyle and clinical factors demonstrated variable influences on semen quality. Smoking showed a clear negative impact on sperm concentration, motility, and morphology, while alcohol consumption exhibited weak positive correlations with sperm count and concentration but reduced motility and normal morphology. Sexually transmitted diseases and trauma negatively influenced selected semen parameters, whereas an active occupational lifestyle showed positive associations with motility and morphology. Overall, the findings emphasize the importance of lifestyle modification in improving semen quality.

Calprotectin, an inflammation-related biomarker in seminal plasma, showed weak negative correlations with most semen parameters, suggesting a marginal association with reduced semen quality. Further studies with larger sample sizes are required to clarify its diagnostic significance.

## Data Availability

The data that support the findings of this study are available from the corresponding author upon request.
